# Tissue tracking under long-horizon occlusions with contrastive learning

**DOI:** 10.1007/s11548-026-03585-4

**Published:** 2026-03-06

**Authors:** Myrto Inglezou, Nikolaos Kegkeroglou, Leonidas Delimpasis, Panagiotis Chatzakos, Antonios Porichis

**Affiliations:** 1 AI Innovation Centre, University of Essex, Wivenhoe Park, Colchester, CO4 3SQ UK; 2 Tech Hive Labs, Iliados 7, 152 32 Halandri, Greece

**Keywords:** Tissue tracking, Self-supervised, Contrastive learning, Long-horizon occlusion

## Abstract

****Purpose**:**

Continuous tracking of soft-tissue regions in minimally invasive surgery is essential for computer-assisted interventions, yet remains highly challenging due to non-rigid tissue deformation, unconstrained endoscopic camera motion, and frequent occlusions caused by surgical instruments. In particular, long-horizon occlusions, where regions of interest exit the field of view and later re-enter from different angles, remain largely unaddressed by existing online tracking methods.

****Methods**:**

We propose a real-time tracking pipeline that integrates dense optical flow for short-term region tracking, monocular visual odometry for camera localization and depth estimation, and a self-supervised template matching module based on contrastive learning for robust tissue re-identification. The template matching component employs a variational encoder trained using time cycle consistency, enabling the learning of deformation-aware visual representations without requiring manual annotations.

****Results**:**

To evaluate our approach, we rely on the public SurgT dataset and a synthetic dataset explicitly designed to feature long-horizon occlusions. The results show that the proposed pipeline maintains stable tracking performance under extended occlusions and viewpoint changes, enabling accurate re-identification of soft-tissue regions after reappearance. The contrastive variational encoder contributes to improved robustness against tissue deformation and appearance variability compared to reconstruction-based or purely geometric baselines.

****Conclusions**:**

Overall, the proposed framework provides a practical, self-supervised solution for long-horizon tissue tracking in minimally invasive surgery, demonstrating promising performance despite current quantitative evaluation being limited to synthetic data due to the lack of suitable real-world benchmarks. The code is available at https://github.com/Essex-AI-Innovation-Centre/cl-ve-tracking

## Introduction

Tracking specific soft-tissue regions during surgery is essential for providing enhanced feedback related to tool-tissue interactions and monitoring high-risk areas [1]. Generally, the process starts with defining a target region in the first frame, followed by estimating its motion throughout the subsequent frames of a surgical video. Existing techniques attempted to deal with this task using rigid assumptions [2, 3], feature descriptors [4], or model-based approaches [5, 6]. Other methods have employed feature-based patch tracking, where appearance features of the template and search region are independently extracted and matched via cross-correlation to locate the position of maximum similarity in the response map [7]. Similar ideas have also been explored in the context of general object tracking using Siamese architectures and correlation filters, which have shown strong performance in rigid or moderately deformable scenes [8, 9]. While effective in structured scenes, these approaches struggle in surgical environments, due to the nature of the tissue itself, which poses a further challenge [10].

To better capture fine-grained motion in intra-operative settings, dense optical flow estimation has been widely adopted for modeling local point-wise displacements and soft tissue recovery [11, 12]. In parallel, SLAM-based methods such as [13–15] have been proposed to jointly estimate camera motion and track soft-tissue regions. While these methods can provide dense geometric context for tissue tracking, they often rely on sparse or feature-rich regions, and their performance can degrade on low-texture tissue surfaces or under strong occlusions. More recently, Tracking Any Point (TAP) techniques [16, 17] estimate optical flow to track the motion of individual points across consecutive frames. By avoiding reliance on object appearance during training, flow-based methods generalize more robustly to previously unseen deformable patterns and different surgical domains.

However, a significant remaining challenge in these settings is long-horizon occlusions, where target regions are obscured by surgical instruments or disappear from the field of view for extended durations. To address this challenge, this work proposes a methodology that utilizes dense optical flow estimation for fine-grained point movements, integrated into a pipeline that incorporates camera localization. This is further augmented by template matching based on contrastive learning, which enhances robustness against severe tissue deformations and texture-less regions. Moreover, the template-matching module facilitates reliable re-identification of the target region when it exits and subsequently re-enters the field of view, ensuring continuous tracking over extended sequences. The main contributions of this work are:

1. Development of a self-supervised template matching method that enables tissue re-identification by learning visual representations through contrastive loss.
2. Implementation of a real-time pipeline for tracking under long-horizon occlusions, that forgoes scene mapping, yet effectively mitigates localization drift via feedback from template matching.
3. Validation of the approach on the public SurgT [18] benchmark and a synthetic dataset specifically designed to evaluate tracking under long-horizon occlusions.

## Related work

Several methods have been proposed for tissue tracking in endoscopic scenes. An online learning-based approach [19] adapts feature representations to remain robust to short-term occlusions, while Giannarou et al. [20] introduce a probabilistic framework for affine-invariant regions. KINFlow [21] uses k-nearest keypoint correspondences to track tissue motion and its extension RING [22], operates in real-time for arbitrary image point tracking. Despite their robustness for continuous visibility, these methods cannot handle long-horizon occlusions when targets exit and later re-enter the frame.

Recent advances in Tracking Any Point (TAP) [16] have demonstrated strong potential for addressing long-horizon occlusions by enabling dense, long-term correspondence estimation across entire video sequences. Methods such as MFT [23] and MFTIQ [24] propose multi-flow tracking with independent quality estimation to improve robustness and efficiency in long-term pixel-level tracking. Building on these developments, TAP methods have recently been extended to the surgical domain [25–27]. Despite their promising results, their point-centric approach lacks contextual information and region-level tracking requires aggregating multiple points that may include outliers or drift. Lastly, for domain-specific adaptation, they require either manual annotation or extensive training with teacher models. More recently, Chen et al. [28] introduced an occlusion-aware extension for surgical point tracking. While this approach effectively maintains sparse correspondences, it remains inherently point-centric and constrained by its reliance on stereo depth cues.

**Fig. 1:**
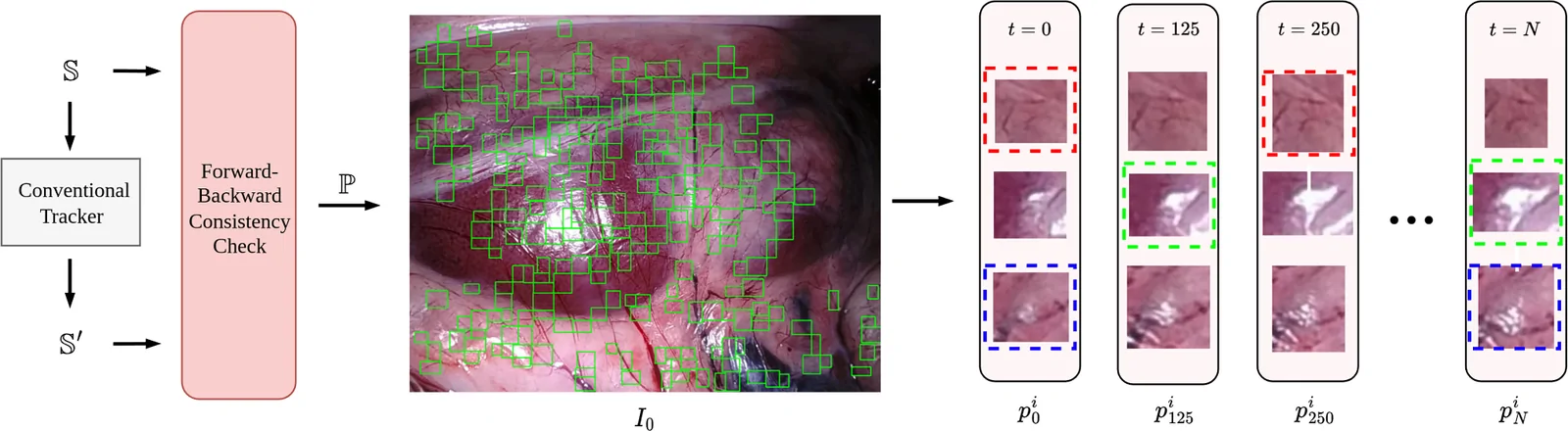
Illustration of the contrastive learning methodology. From left to right: the time cycle consistency technique extracts a set of patches from the initial frame ($I_0$), serving as inputs for contrastive learning. The patches with same colored dashed boxes represent examples of positive pairs, while patches with different colors represent negative pairs

In the context of surgical scene reconstruction, SLAM-based approaches have been developed to integrate localization and tissue tracking [3]. Works such as [4], account for soft-tissue deformation, but rely on keypoint-based feature matching (e.g., SIFT [29]), which struggles on texture-less tissue and under distortions or lighting variations. Neural rendering methods [30] produce detailed 3D reconstructions, but operate offline and depend on tool masking from pre-trained models. Online approaches such as [11] enable real-time surface mapping and point-wise tracking, remaining robust to tool occlusions and camera motion, yet still require stereo input.

Template matching has long been a fundamental strategy for localization and tracking. Recent works include QATM [31], which incorporates quality-aware similarity estimation for reliable patch matching, and Self-TM [32] that introduces a self-supervised foundation model, claiming zero-shot generalization capability on dense template matching. Despite their general robustness, they are not specifically tailored for surgical scenes. In contrast, Ada-Tracker [12] utilizes adaptive-template matching to improve robustness in deformable tissue tracking, but does not explicitly address long-horizon occlusions.

## Methodology

In this section, we detail the components that comprise the proposed pipeline. First, the methodology on optical flow, camera localization, and 3D tracking of the target area. Next, we present the contrastive learning framework for template matching, followed by the integration of these components into a coherent tracking system.

### Optical flow

We employ NeuFlow-V2 [33] to estimate dense optical flow, which achieves high accuracy in real-world datasets with lower computational overhead compared to other state-of-the-art optical flow methods. Given a pair of consecutive RGB images $\textbf{I}_{t-1}$ and $\textbf{I}_t$, the optical flow model computes the dense flow field $\textbf{X}_t$ at timestep *t*. For a target area with (*u*, *v*) as the centroid pixel coordinates and (*w*, *h*) as the width and height of the corresponding bounding box *b*, tracking is achieved by moving the box using the median flow vector:

1 $$\begin{aligned} \tilde{\textbf{x}}_{t}^{b}  &   = \text {median} \{ \textbf{X}_{t}[i,j] \; \vert \; u-h/2 \nonumber \\  &   \le i<u+h/2, \; v-w/2\le j<v+w/2 \} \end{aligned}$$

### Camera localization

To handle both long-horizon and in-frame occlusions, we utilize VOLDOR [34], a dense indirect visual odometry method, that operates in real-time on monocular video. In the proposed pipeline, VOLDOR takes a sequence (batch) of size *B* of optical flow fields $$\mathbb {X}_{n:n'} = \{\textbf{X}_t \mid n < t \le n' \},$$ as input and jointly estimates scene depth map $$\textbf{D}_{n}$$ at keyframe t=n and the 6-DoF relative camera motion $$\textbf{T}_{n,n'} \in SE(3)$$, from *n* to $n^{\prime }$ through a probabilistic model. Since it operates in batches, we adopt the concept of distinct keyframes throughout the video, rather than making predictions on successive timesteps.

**Fig. 2:**
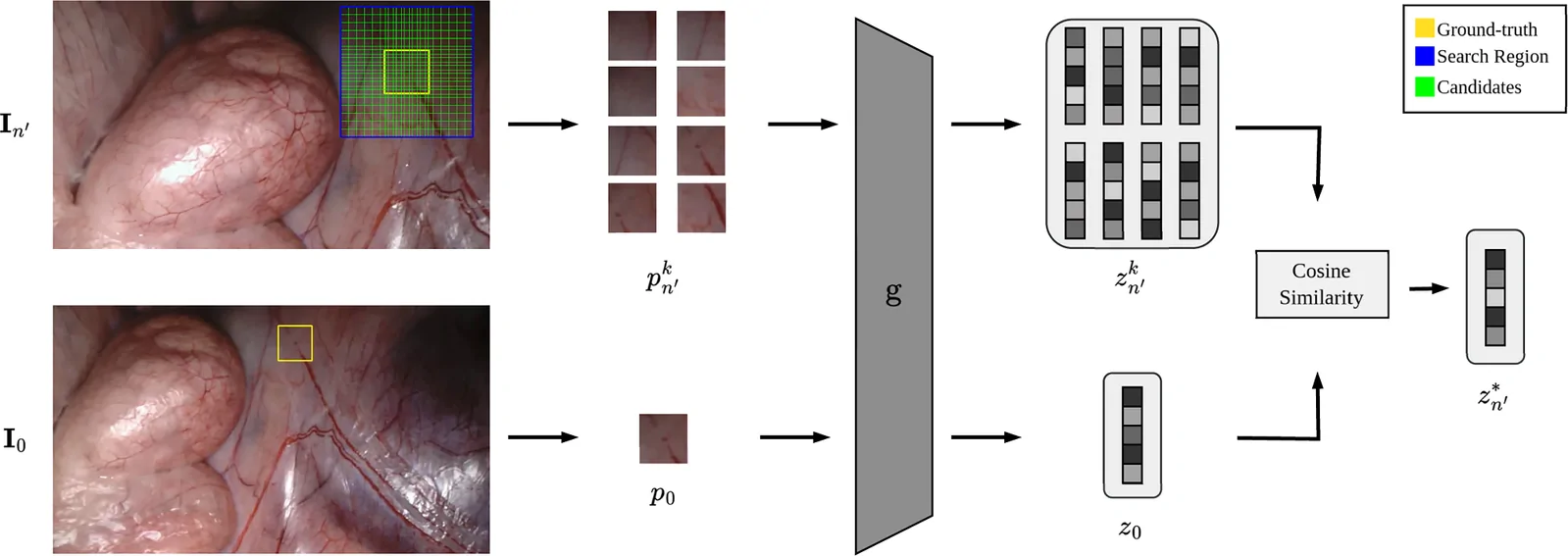
Overview of the template matching for handling the scenario of an occlusion

### 3D tracking

During occlusion, i.e., while the target area either remains outside the frame or is obscured in-frame, 3D tracking is performed to continuously provide a location estimate until re-identification. Given the camera intrinsic matrix $$\textbf{K}$$, the estimated depth map $$\textbf{D}_{n}$$, the camera motion $$\textbf{T}_{n,n'}$$, and the area’s centroid pixel coordinates $$(u_{n},v_{n})$$ in the latest keyframe *n*, where it was within the frame, we obtain its world coordinates at keyframe $n^{\prime }$:

2 $$\begin{aligned} [X_{n'}\;Y_{n'}\;Z_{n'}]^{T} = \textbf{T}_{n,n'} \left( \textbf{D}_{n}[u_n,v_n]\cdot \textbf{K}^{-1}[u_n\;v_n\;1]^{T} \right) \end{aligned}$$

Then, the area’s centroid pixel coordinates at keyframe $n^{\prime }$ can be derived, by simply projecting on the image plane:

3 $$\begin{aligned} [u_{n'}\;v_{n'}]^{T} = \textbf{K}\;\left[ \frac{X_{n'}}{Z_{n'}}\;\frac{Y_{n'}}{Z_{n'}}\right] ^{T} \end{aligned}$$

### Template matching

To address drift caused by accumulated localization errors, a refinement of the coarse 3D tracking estimate is necessary. For this purpose, we exploit a Variational Autoencoder (VAE) formulation [35] and employ its encoder within a contrastive learning framework. Contrary to conventional training of such autoencoder models, the proposed approach leverages the probabilistic latent representation learned by the variational encoder and explicitly discards the decoder during inference. The motivation for this choice is that modeling latent embeddings as distributions, rather than deterministic vectors, provides an inherent regularization mechanism that improves robustness to soft-tissue deformations, appearance ambiguity, and uncertainty induced by occlusions. Within the contrastive learning regime, the encoder is encouraged to produce compact and consistent latent distributions for the same tissue region across time, while maintaining separation from other regions. Training is conducted in a self-supervised manner, as shown in Fig. 1. We refer to our model as Contrastive Learning Variational Encoder (CL-VE), as the VAE’s decoder is not utilized. The model’s core principle is to learn visual representations of small patches (image regions) in the latent space by minimizing the projection distance between a template patch and its future form at later frames, while maximizing the distance from other patches, thereby facilitating robust tissue re-identification under long-horizon occlusions.

To train the model, we utilize a straightforward and zero-annotation, yet effective technique, which relies on the concept of time cycle consistency. The process begins with Shi-Tomasi [36] corner detection, which identifies a set $\mathbb {S}_{0}$ of *M* total keypoints in the first frame $I_{0}$ of an input video with *N* total frames. These keypoints are tracked forwards using the pyramidal Lucas-Kanade [37], an extremely lightweight, widely available off-the-shelf sparse optical flow algorithm that requires no manual supervision, resulting in $\mathbb {S}=\{s_{t}^{i} \; \vert \; 0 \le t< N,\; 0 \le i < M\}$. To ensure tracking reliability, we also perform this step backwards, starting from $\mathbb {S}_{N}$, thus producing $\mathbb {S^{\prime }}$, and apply a tracking consistency check for every successfully tracked keypoint. More specifically, we filter out inconsistencies by preserving keypoints that satisfy a Euclidean distance condition: $||s_t^i-s_{t}^{i\prime }||<\tau$, where τ denotes a fixed distance threshold. The final step for data collection involves the generation of a set of *Q* total patches $\mathbb {P}=\{p_{t}^{k} \; \vert \; 0 \le t< N,\; 0 \le k < Q\}$ with each patch region defined from a point quartet $(s_t^{i_1}, s_t^{i_2}, s_t^{i_3}, s_t^{i_4})$ that complies with spatial constraints, to eliminate overlaps and impose a specific bounding box size range.

Given a patch $p_{t}^{k}$, the variational encoder $\textrm{g}(\cdot )$ outputs two vectors, $\textbf{g}_\mu (p_t^{k})$ and $\textbf{g}_\sigma (p_t^{k})$, which are treated as the mean and diagonal covariance matrix of a normal distribution. An embedding of $p_t^{k}$ is sampled from the captured latent space:

4 $$\begin{aligned} \textbf{z}_{t}^{k}=\textbf{g}_\mu (p_t^{k}) + \mathbf {\epsilon } \; \odot \; \textbf{g}_\sigma (p_t^{k}) \end{aligned}$$

where $\mathbf {\epsilon }\sim \mathcal {N}(0,\textbf{I})$ and ⊙ denotes the element-wise product.

**Fig. 3:**
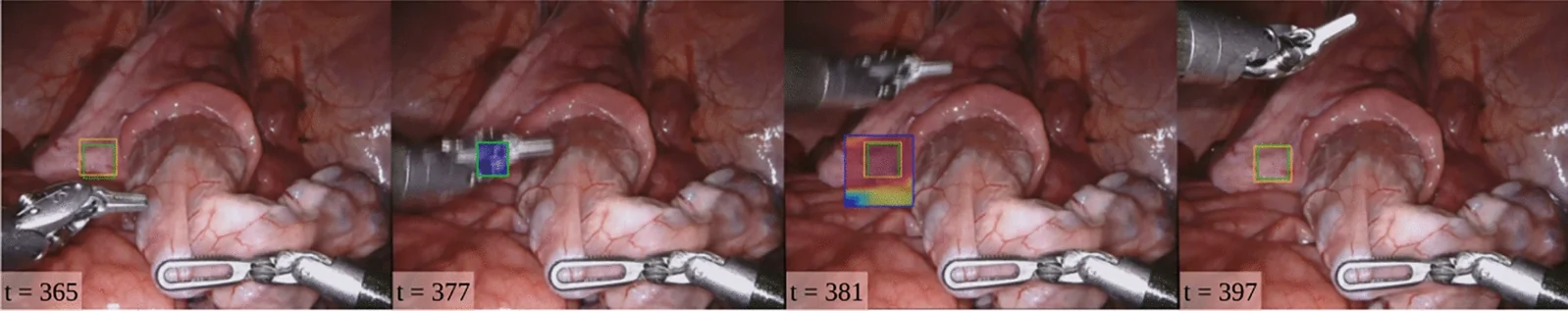
Example of tool interference handling using the proposed CL-VE–based template matching. The heatmap overlay highlights cosine similarity values for candidate patches, with scores increasing from blue to red

For an input video, each generated patch $$p_{t}^{k}$$ is assigned a positive example $$p_{t'}^{k}$$ at a random timestep $t^{\prime }$ independently chosen for each patch, forming a positive embedding pair $$(\textbf{z}_t^k, \textbf{z}_{t'}^k)$$ using Eq. (4). Sequentially, we create batches of positive embedding pairs by random sampling, where each batch $$\mathbb {B}$$ is defined as $$\{(\textbf{z}_t^l, \textbf{z}_{t'}^l) \; \vert \; 0 \le l < Q\}$$. In similar fashion to [38], we do not explicitly define negative pairs. Instead, for each embedding $$\textbf{z}^{k}_{t}$$ we treat $$\{\textbf{z}^{l}_{t},\textbf{z}^{l}_{t'}\}_{l\ne k}$$ within a batch as negative examples. The contrastive prediction task for a batch $$\mathbb {B}$$ aims to identify $$\textbf{z}_{t'}^{k}$$ for a given $$\textbf{z}_{t}^{k}$$ among the negative examples, ensuring that the model learns to distinguish between different tissue regions. The loss function for a positive pair is defined as:

5 $$\begin{aligned} \ell (\textbf{z}_t^k, \textbf{z}_{t'}^k) = -\log \frac{\exp (\text {sim}(\textbf{z}_t^k, \textbf{z}_{t'}^k) / \tau )}{\sum _{i\in \{t,t'\}} \sum _{j=1}^{Q} 1\!\!1_{[i \ne k]} \exp (\text {sim}(\textbf{z}_t^k, \textbf{z}_{i}^j) / \tau )} \end{aligned}$$

where $\text {sim}(\cdot , \cdot )$ denotes the cosine similarity function, $1\!\!1_{[j\ne l]}\in \{0, 1\}$ is an indicator function evaluating to 1 iff j≠l and τ denotes a temperature parameter. The final loss is computed as the arithmetic mean of all positive pairs in the batch:

6 $$\begin{aligned} \mathcal {L}_{\mathbb {B}} = \frac{1}{2Q} \sum _{l=1}^{Q} \left[ \ell (\textbf{z}_t^l, \textbf{z}_{t'}^l) + \ell (\textbf{z}_{t'}^l, \textbf{z}_{t}^l) \right] \end{aligned}$$

### Algorithm integration

An outline of the proposed workflow is provided next. Initially, $$\textrm{g}$$ produces the embedding $$z_{0}$$ of the target area in the first frame $$\textbf{I}_{0}$$. The optical flow model tracks the corresponding bounding box $$b_{0}$$ in the 2D plane using Eq. (1) as long as it remains within the camera’s field of view. In parallel, the dense flow fields $$\textbf{X}_t$$ computed in batches $$\mathbb {X}_{n:n'}$$ at every timestep *t*, are forwarded to the camera localization module, which subsequently estimates $$\textbf{T}_{n,n'}$$ and $$\textbf{D}_{n}$$. To enhance tracking, the dimensions $$(w_n,h_n)$$ of $$b_n$$ are adjusted according to the estimated pixel-depth $$\textbf{D}_{n}[u_n,v_n]$$. Additionally, the CL-VE extracts the target area’s embedding to compare it with the template one and perform similarity check for in-frame occlusion. In such case, or when the target area exits the frame, we track it in 3D using Eq. (2) and continuously monitor its projection from Eq. (3), on whether it lies within the frame. Once the system determines the target’s re-appearance at keyframe $n^{\prime }$ with centroid coordinates $$(u_{n'},v_{n'})$$, a search region is established by defining a scaling factor λ to account for localization error:

7 $$\begin{aligned} S_{n'} = \left\{ \textbf{I}_{n'}[i,j] \;\bigg |\; \begin{array}{c} u_{n'}-h_{n'}\sqrt{\lambda }\le i<u_{n'}+h_{n'}(1+\sqrt{\lambda }) \\ v_{n'}-w_{n'}\sqrt{\lambda }\le j<v_{n'}+w_{n'}(1+\sqrt{\lambda }) \end{array} \right\} \end{aligned}$$

The search region is then divided into a grid of candidate patches $\{p_{n'}^{k}\}_{k=1}^{C_{n'}}$, by sliding a bounding box across the image, in order to perform template matching, as illustrated in Fig. 2.

The total number of candidates is:

8 $$\begin{aligned} C_{n'}=\left( \left\lfloor h_{n'} (\sqrt{\lambda } - 1)/s \right\rfloor + 1 \right) \cdot \left( \left\lfloor w_{n'} (\sqrt{\lambda } - 1)/s \right\rfloor + 1 \right) \end{aligned}$$

where *s* denotes the stride and $(w_{n'},h_{n'})$ are the dimensions of the target area, according to estimated pixel-depth $\textbf{D}_{n'}[u_{n'},v_{n'}]$. The candidate patches are fed into the CL-VE, which produces $\{z_{n'}^{k}\}_{k=1}^{C_{n'}}$ and identifies the embedding $z_{n'}^{*}$, that exhibits the highest cosine similarity to the template embedding $z_{0}$:

9 $$\begin{aligned} z_{n'}^{*} = \max \{\text {sim}(z_{0},z_{n'}^{k}) \; \vert \; 0 < k \le C_{n'}\} \end{aligned}$$

**Table 1:** Quantitative comparison of template matching and end-to-end pipelines

Evaluation Task	Method	SurgT dataset		Synthetic dataset	
		$\text {Acc}_{\text {2D}} \uparrow$	$\text {Err}_{\text {2D}}\% \downarrow$	$\text {Acc}_{\text {2D}} \uparrow$	$\text {Err}_{\text {2D}}\% \downarrow$
Template matching	SIFT [29]	0.474	4.35	0.364	1.25
	QATM [31]	0.510	2.70	0.311	1.49
	Self-TM [32]	0.498	2.47	0.478	1.06
	CL-VE	**0**.**618**	**2**.**10**	**0**.**545**	**0**.**86**
End-to-End tracking	MFT [23]	0.519	1.85	0.166	4.72
	MFTIQ [24]	0.418	4.29	0.213	3.38
	CoTracker3 [41]	0.715	**0**.**64**	0.545	1.05
	Ours	**0**.**721**	1.09	**0**.**653**	**0**.**49**

Best results are noted with bold and the second best with underlining

The template matching process is repeated for *L* keyframes, resulting in a set of final matching candidates $\{z_{n}^{*}\}_{L}$. The final matching decision follows the same identification protocol as previously described, by selecting the most similar to $z_{0}$. The corresponding bounding box is then moved to the current keyframe *n*, by utilizing the essential pre-computed intermediate dense flows from $\mathbb {X}_{(n'+1):(n'+L)}$. Subsequently, the optical flow model continues the tracking process of the bounding box $b_{n}$ as in Eq. (1).

Upon completion of the template matching procedure, the centroid pixel coordinates $(u_{n},v_{n})$ are employed as feedback to refine the camera localization estimate, using Eq. (2). This refinement step is crucial for addressing subsequent exits of the target area, as it mitigates drift caused by the accumulation of potential localization errors. In the cases of significant occlusions due to tissue overlap or tool interference during in-frame tracking with optical flow, the CL-VE detects low similarity between the tracked and template patch and effectively handles such scenarios, as shown in Fig. 3. The dense modules of optical flow and depth estimation are crucial components of the pipeline, supporting both localization refinement and in-frame occlusion detection.

**Fig. 4:**
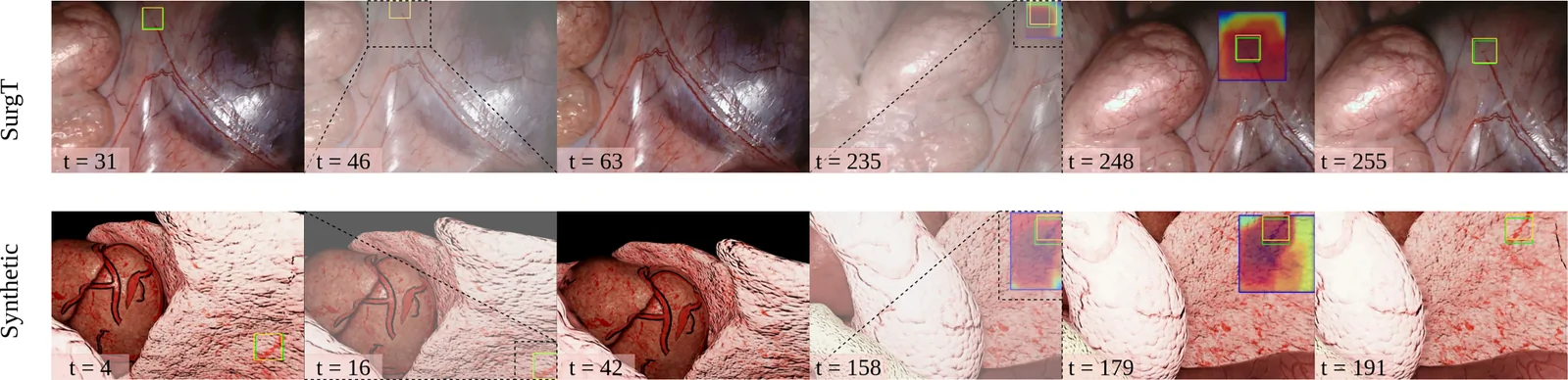
Visual examples of long-horizon occlusions in both selected datasets. From left to right: The target area is initially visible, then moves out of the field of view, and after arbitrary camera movements, it reappears from a different angle. Next, the CL-VE makes the final matching decision

## Experiments

**Datasets** To validate the proposed solution, we rely on the public SurgT dataset [18], which contains 157 endoscopic videos (125 for training, 12 for validation, and 20 for testing). Furthermore, we acquire a synthetic dataset, since SurgT includes a limited number of long-horizon occlusions and its evaluation protocol re-initializes bounding boxes upon reappearance. To generate the synthetic dataset, we utilize the human anatomical organs provided by the ORBIT-Surgical [39] framework, a surgical robot simulation environment with photorealistic rendering built on top of NVIDIA Isaac Sim [40]. We also integrated a monocular camera and an internal light source to mimic the endoscope. The resulting dataset consists of 115 videos (78 for training, 22 for validation, and 15 for testing) at 1280×720 resolution with 80% of the testing episodes featuring long-horizon occlusions.

**Implementation details** All experiments were conducted in PyTorch on an NVIDIA RTX 3060 GPU. The CL-VE was trained using the Adam optimizer for 50 iterations, with a learning rate of $3\mathrm {\times }10^{-4}$, requiring approximately 15 min per epoch and the model with the best validation performance was retained. Inference achieved ∼15 FPS (135 ms per keyframe) with VOLDOR batch size B=2. Input patches were resized to 16×16, and template matching ran efficiently (10 ms) as candidate embeddings (Eq. 8) were extracted in a single forward pass. The normalized similarity threshold for the occlusion check was set to 0.7 and the search region scaling to λ=0.5. Regarding CL-VE, stride was fitted to s=6 and the number of final matching decision keyframes to L=5. For SIFT, the contrast threshold was fixed at $\tau _{c}=0.02$ and the edge threshold at $\tau _{e}=10$. These parameters were selected after a grid search, over $s\in [2,10], \; L\in [1,5], \; \tau _{c}\in [0.02, 0.06], \; \tau _{e}\in [8,14]$, with steps of $2, \;1, \;0.01,\;$ and 2, respectively.

**Fig. 5:**
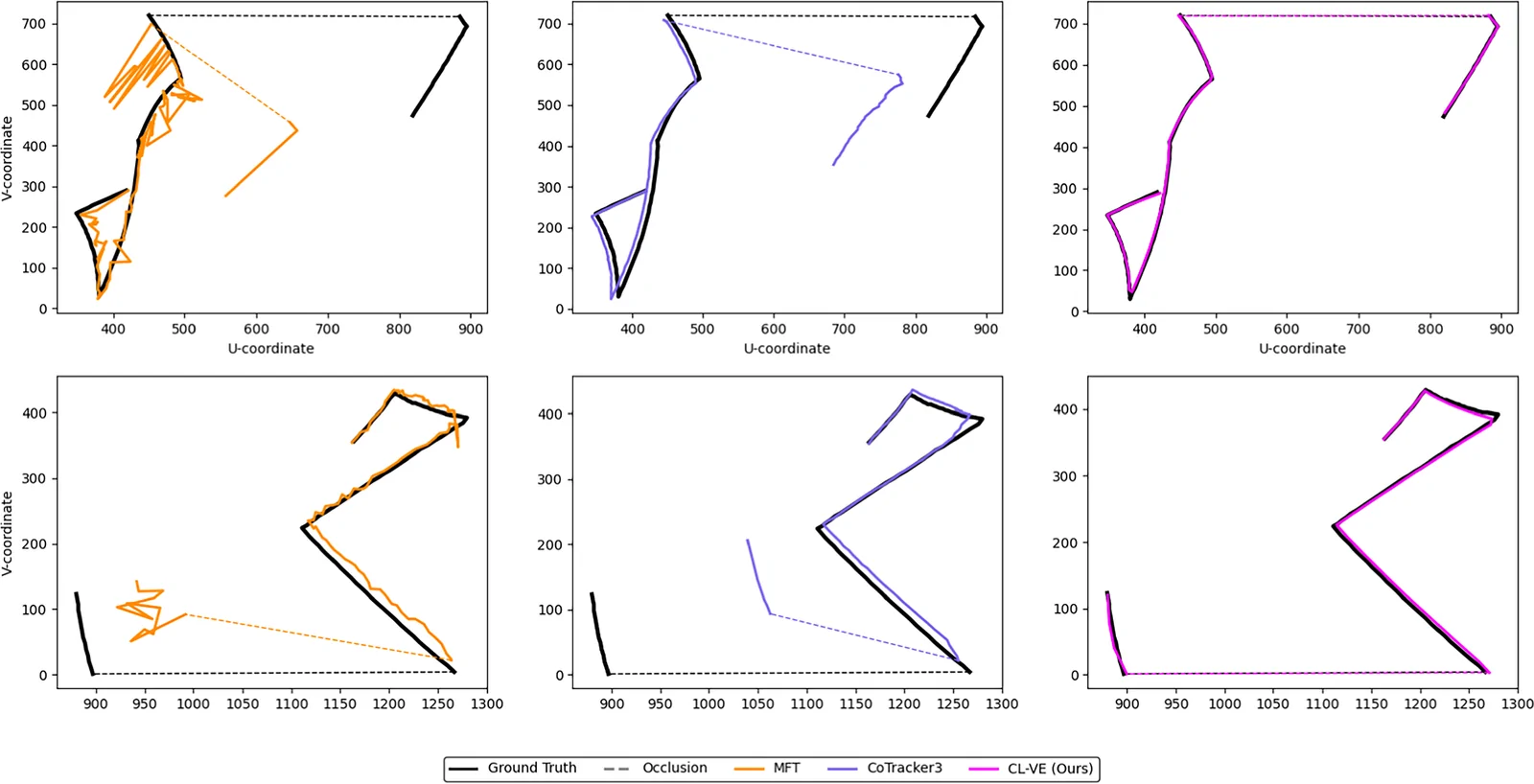
Qualitative comparison of tracking trajectories during long-horizon occlusion scenarios of the synthetic dataset. Ground-truth trajectories are shown in black, dashed segments indicate frames where the target patch is fully occluded. The colored plots illustrate trajectories in pixel coordinates (*u*, *v*) for three methods: MFT (orange), CoTracker3 (blue), and the proposed CL-VE (magenta)

**Results** We conduct experiments with two main objectives. First, we evaluate the CL-VE in isolation for the task of template matching, i.e., re-identifying a target tissue area within the established search region. This evaluation isolates the contribution of the learned visual representation and allows direct comparison against existing template matching approaches, including SIFT [29], QATM [31], and the recent Self-TM [32]. Second, we evaluate the proposed pipeline alongside state-of-the-art trackers such as MFT [23], its extension MFTIQ [24], and CoTracker3 [41], in order to quantify overall tracking performance. We report the monocular metrics of average IoU between the ground truth and predicted bounding boxes ($\text {Acc}_{\text {2D}}$), along with the percentage of 2D pixel error ($\text {Err}_{\text {2D}}\%$), computed as the mean Euclidean distance between ground truth and predicted centroids, normalized by the frame diagonal. The resulting metrics cannot be directly compared to the SurgT benchmark as its evaluation protocol re-initializes the bounding box upon reappearance, which implies that cases of long-horizon occlusions are not considered. Additionally, our metrics are not calculated for all video frames, since our method operates on frame batches, as described in Sects. 3.2 and 3.3.

To evaluate the CL-VE on the template matching task, the candidate patches from the established search region (Eq. (7)) are given as input in every keyframe. The model then provides the prediction corresponding to the patch with the highest similarity, as detailed in Sect. 3.5. For SIFT, the prediction is the bounding box with its centroid being the median of the matched feature points, after outliers are rejected using a distance condition, and its dimensions adjusted according to estimated depth. In the end-to-end tracking task, the predictions of MFT, MFTIQ and CoTracker3 are defined as the bounding box that encloses all tracked points, which were queried as the target in the initial frame.

Table 1 presents a quantitative comparison of template matching and end-to-end tracking methods across the SurgT and synthetic datasets. For the template matching task, CL-VE achieves the highest $\text {Acc}_{\text {2D}}$ and the lowest $\text {Err}_{\text {2D}}\%$ on both datasets, surpassing SIFT and QATM, as well as the Self-TM model. In the context of end-to-end tracking, our proposed pipeline, which incorporates CL-VE as a key component, achieves the best overall performance. On the SurgT dataset, our method yields a 0.6% improvement in $\text {Acc}_{\text {2D}}$ over CoTracker3, with only a 0.45% rise in $\text {Err}_{\text {2D}}$. Notably, depth estimation influences the size of the bounding box prediction, which significantly impacts IoU ($\text {Acc}_{\text {2D}}$) but does not affect pixel error ($\text {Err}_{\text {2D}}\%$). Additionally, on the synthetic dataset, the presented approach significantly outperforms all competing approaches across all metrics, achieving both higher 2D accuracy and lower 2D error. Lastly, the lower-resolution videos in SurgT amplify 2D pixel error percentage, meaning that improvements in accuracy do not necessarily coincide with reductions in pixel error, and vice versa.

We observe that the proposed method effectively handles long-horizon occlusions, a capability that is particularly evident on the synthetic dataset, where occlusion scenarios are more frequent, compared to SurgT, which contains only a few such cases. Alongside the evident performance gain, Fig. 4 illustrates representative examples demonstrating how CL-VE successfully addresses these challenges on both datasets. Additionally, Fig. 5 presents tracking trajectory comparisons among the evaluated methods on the synthetic dataset.

**Table 2:** Ablation study of the template matching component

Method	SurgT dataset			Synthetic dataset		
	$\text {Acc}_{\text {2D}} \uparrow$	$\text {Err}_{\text {2D}}\% \downarrow$	$\text {Src}_{\text {2D}}\% \downarrow$	$\text {Acc}_{\text {2D}} \uparrow$	$\text {Err}_{\text {2D}}\% \downarrow$	$\text {Src}_{\text {2D}}\% \downarrow$
Rec-VAE	0.324	5.19		0.254	1.66	
CL-CNN	0.504	2.92	4.05	0.439	1.16	1.52
CL-VE	**0**.**618**	**2**.**10**		**0**.**545**	**0**.**86**	

**Ablation study** We conduct an ablation analysis to evaluate the individual contributions of the variational architecture and the contrastive learning objective within the template matching component. Specifically, we compare the proposed CL-VE against two variants: Rec-VAE, trained solely with a conventional reconstruction loss, and CL-CNN, which employs the same encoder architecture but lacks the variational probabilistic part. As shown in Table 2, the Rec-VAE exhibits the lowest performance across all metrics, with an $\text {Acc}_{\text {2D}}$ of 0.324 on SurgT and 0.254 on the synthetic dataset. This confirms that pixel-level reconstruction is insufficient for capturing the complex non-rigid deformations inherent in surgical scenes. While the CL-CNN shows improved results by utilizing contrastive loss, it still underperforms compared to the CL-VE, particularly in the synthetic dataset where it achieves an $\text {Acc}_{\text {2D}}$ of 0.439 versus our 0.545. These results suggest that the variational part is crucial. Therefore, we may conclude that by treating embeddings as distributions rather than deterministic points, the model effectively regularizes the latent space and provides a principled mechanism for handling uncertainty during long-horizon occlusions.

Furthermore, to decouple the contributions of 3D tracking via camera localization and template matching, we conduct an experiment in which the template matching component is not utilized, and tracking is performed using only the camera localization module. In this setting, we report the percentage of 2D search error ($\text {Src}_{\text {2D}}\%$), computed as the mean Euclidean distance between the ground truth and search region’s centroids, normalized by the frame diagonal. As shown in Table 2, using only camera localization results in an $\text {Src}_{\text {2D}}$ of 4.05% on SurgT and 1.52% on the synthetic dataset. This metric is identical for all three encoder variants (Rec-VAE, CL-CNN, and CL-VE) when template matching is disabled, providing a common baseline. While Rec-VAE is slightly worse than plain 3D tracking, the CL variants significantly improve performance when template matching is enabled. Specifically, the CL-VE achieves an $\text {Err}_{\text {2D}}$ of 2.10% on SurgT and 0.86% on the synthetic dataset. These results indicate that while 3D tracking provides a coarse estimate of target motion, appearance-based template matching is critical for accurately re-identifying the target area, particularly under long-horizon occlusions.

## Conclusion

We have introduced a pipeline, with self-supervised contrastive learning for template matching to track soft tissue in the intraoperative field. Experiments on both the SurgT and the synthetic dataset demonstrate that our method achieves high tracking accuracy, and significantly improves performance in long-horizon occlusion scenarios compared to existing methods. Unlike methodologies that leverage pre-trained models on general computer vision datasets, which do not closely match surgical scenes, our approach can be trained directly on any custom dataset without annotations or additional supervision. A current limitation of this work is that long-horizon reappearance scenarios are primarily evaluated on synthetic data, due to the lack of public surgical benchmarks that capture such events. Extending validation to phantom-based setups that mimic real intraoperative conditions, or to actual surgical scenarios that include long-horizon occlusions, represents an important direction for future work.
